# (1*R*,2*S*,3*R*,5*S*)-5-(1*H*-Benzimidazol-2-yl)cyclo­hexane-1,2,3,5-tetraol mono­hydrate

**DOI:** 10.1107/S1600536809037957

**Published:** 2009-09-26

**Authors:** Ying Cai

**Affiliations:** a Ordered Matter Science Research Center, Southeast University, Nanjing 211189, People’s Republic of China

## Abstract

In the crystal structure of the title compound, C_13_H_16_N_2_O_4_·H_2_O, inter­molecular N—H⋯O, O—H⋯O and O—H⋯N hydrogen bonds form an extensive three-dimensional network, consolidating the crystal packing. The cyclo­hexane ring adopts a chair conformation.

## Related literature

For the crystal structures of related compounds, see: Li *et al.* (1998 ▶); Gallagher *et al.* (2001 ▶); Howarth & Hanlon (2001 ▶); Huang *et al.* (2003 ▶); Kazak *et al.* (2006 ▶). For ring-puckering parameters, see: Cremer & Pople (1975 ▶).
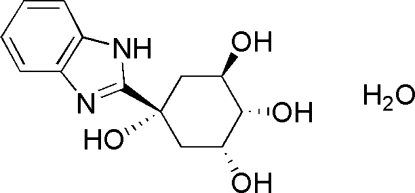


## Experimental

### 

#### Crystal data

C_13_H_16_N_2_O_4_·H_2_O
*M* *_r_* = 282.29
Orthorhombic, 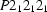

*a* = 8.9684 (14) Å
*b* = 9.4809 (15) Å
*c* = 15.278 (4) Å
*V* = 1299.0 (4) Å^3^
*Z* = 4
Mo *K*α radiation
μ = 0.11 mm^−1^
*T* = 293 K
0.30 × 0.30 × 0.30 mm

#### Data collection

Rigaku Mercury CCD diffractometer
Absorption correction: multi-scan (*CrystalClear*; Rigaku, 2005 ▶) *T*_min_ = 0.817, *T*_max_ = 0.906
12121 measured reflections
1716 independent reflections
1646 reflections with *I* > 2σ(*I*)
*R*_int_ = 0.030

#### Refinement

*R*[*F* ^2^ > 2σ(*F* ^2^)] = 0.033
*wR*(*F* ^2^) = 0.079
*S* = 1.08
1716 reflections
253 parameters
All H-atom parameters refined
Δρ_max_ = 0.14 e Å^−3^
Δρ_min_ = −0.18 e Å^−3^

### 

Data collection: *CrystalClear* (Rigaku, 2005 ▶); cell refinement: *CrystalClear*; data reduction: *CrystalClear*; program(s) used to solve structure: *SHELXS97* (Sheldrick, 2008 ▶); program(s) used to refine structure: *SHELXL97* (Sheldrick, 2008 ▶); molecular graphics: *SHELXTL/PC* (Sheldrick, 2008 ▶); software used to prepare material for publication: *SHELXL97*.

## Supplementary Material

Crystal structure: contains datablocks I, global. DOI: 10.1107/S1600536809037957/bx2237sup1.cif

Structure factors: contains datablocks I. DOI: 10.1107/S1600536809037957/bx2237Isup2.hkl

Additional supplementary materials:  crystallographic information; 3D view; checkCIF report

## Figures and Tables

**Table 1:** Hydrogen-bond geometry (Å, °)

*D*—H⋯*A*	*D*—H	H⋯*A*	*D*⋯*A*	*D*—H⋯*A*
N2—H6⋯O1^i^	0.90 (3)	2.11 (3)	2.983 (2)	164 (2)
O3—H12⋯O5^ii^	0.83 (3)	2.00 (3)	2.831 (2)	176 (3)
O4—H13⋯O2	0.89 (3)	1.87 (3)	2.660 (2)	148 (3)
O2—H14⋯O5^iii^	0.84 (3)	1.93 (3)	2.743 (2)	163 (3)
O1—H15⋯O3^iv^	0.86 (3)	2.21 (3)	3.066 (2)	172 (3)
O5—H17⋯O4^v^	0.88 (3)	1.96 (4)	2.827 (2)	169 (3)
O5—H18⋯N1	0.93 (4)	1.81 (4)	2.740 (2)	176 (3)

Symmetry codes: (i) 
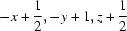
; (ii) 
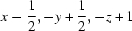
; (iii) 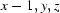; (iv) 
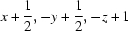
; (v) 
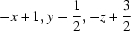
.

**Fig. 1.**
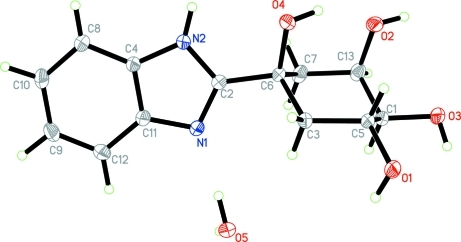
A view of compound (1) with the atomic numbering scheme. Displacement ellipsoids were drawn at the 30% probability level.

**Fig. 2.**
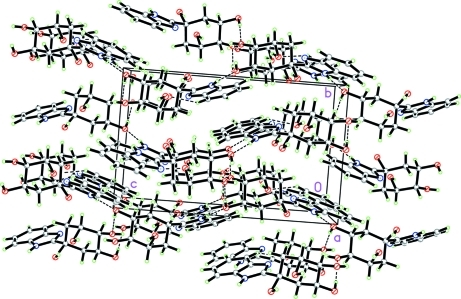
The crystal packing of the title compound viewed along the a axis and all water molecules were omitted for clarity.

## supplementary crystallographic information

### Comment

It has been generally accepted that benzimidazole systems continue to attract much attention due to applications in : chemical synthesis, structural science, applied biological and coordination chemistry (Gallagher *et al.*, 2001; Huang *et al.*, 2003; Kazak *et al.*, 2006). We report here the crystal structure of the title compound (Fig.1). The cyclohexane ring adopt a chair conformation as shown by the Cremer and Pople (1975) puckering parameters [Q_T_= 0.573 (2) Å, θ =174.2 (2) ° and

φ=136 (2)°] and has the same configuration as the cyclohexane ring of (1*S*,3*R*,4*S*,5*R*)-1,3,4,5-tetrahydroxycyclohexanecarboxylic acid, used as started material . The crystal is stabilized by hydrogen bonds of N—H···O, O—H···O and hydrogen bond of O—H ···N, resulting in an extensive three-dimensional network (Fig. 2).

### Experimental

(1*S*,3*R*,4*S*,5*R*)-1,3,4,5-tetrahydroxycyclohexanecarboxylic acid (0.02 mol, 3.84 g) and benzene-1,2-diamine (0.02 mol, 2.16 g) were dissolved in 5.5 N HCl (20 ml) at 110°C with stirring for 24 h at 110°C. After the solution was cooled to room temperature, the pH was adjusted to 8–9 with NaOH solution. The product formed was filtered, washed with ethanol and dried. Further purification was done by recrystallization from methanol. Single crystals suitable for X-ray analysis were obtained with about 50% yield.

### Refinement

All H atoms were located in a difference Fourier map and refined isotropically; the C-H and O-H bond distances are in the ranges 0.95 (2)-1.04 (3) and 0.83 (3)-0.93 (4) Å; N-H = 0.90 (3) Å. Friedel pairs were merged.

### Crystal data

**Table:**

C_13_H_16_N_2_O_4_·H_2_O	*F*(000) = 600
*M**_r_* = 282.29	*D*_x_ = 1.443 Mg m^−^^3^
Orthorhombic, *P*2_1_2_1_2_1_	Mo *K*α radiation, λ = 0.71073 Å
Hall symbol: P 2ac 2ab	Cell parameters from 3450 reflections
*a* = 8.9684 (14) Å	θ = 2.6–27.4°
*b* = 9.4809 (15) Å	µ = 0.11 mm^−^^1^
*c* = 15.278 (4) Å	*T* = 293 K
*V* = 1299.0 (4) Å^3^	Prism, pink
*Z* = 4	0.30 × 0.30 × 0.30 mm

### Data collection

**Table:**

Rigaku Mercury CCD diffractometer	1716 independent reflections
Radiation source: fine-focus sealed tube	1646 reflections with *I* > 2σ(*I*)
graphite	*R*_int_ = 0.030
Detector resolution: 13.6612 pixels mm^-1^	θ_max_ = 27.5°, θ_min_ = 2.6°
ω scans	*h* = −11→11
Absorption correction: multi-scan (*CrystalClear*; Rigaku, 2005)	*k* = −12→12
*T*_min_ = 0.817, *T*_max_ = 0.906	*l* = −18→19
12121 measured reflections	

### Refinement

**Table:**

Refinement on *F*^2^	Secondary atom site location: difference Fourier map
Least-squares matrix: full	Hydrogen site location: inferred from neighbouring sites
*R*[*F*^2^ > 2σ(*F*^2^)] = 0.033	All H-atom parameters refined
*wR*(*F*^2^) = 0.079	*w* = 1/[σ^2^(*F*_o_^2^) + (0.0412*P*)^2^ + 0.2437*P*] where *P* = (*F*_o_^2^ + 2*F*_c_^2^)/3
*S* = 1.08	(Δ/σ)_max_ < 0.001
1716 reflections	Δρ_max_ = 0.14 e Å^−^^3^
253 parameters	Δρ_min_ = −0.18 e Å^−^^3^
0 restraints	Extinction correction: *SHELXL97* (Sheldrick, 2008)
Primary atom site location: structure-invariant direct methods	Extinction coefficient: 0.0476 (15)

### Special details

**Table:**

Geometry. All e.s.d.'s (except the e.s.d. in the dihedral angle between two l.s. planes) are estimated using the full covariance matrix. The cell e.s.d.'s are taken into account individually in the estimation of e.s.d.'s in distances, angles and torsion angles; correlations between e.s.d.'s in cell parameters are only used when they are defined by crystal symmetry. An approximate (isotropic) treatment of cell e.s.d.'s is used for estimating e.s.d.'s involving l.s. planes.
Refinement. Refinement of *F*^2^ against ALL reflections. The weighted *R*-factor *wR* and goodness of fit *S* are based on *F*^2^, conventional *R*-factors *R* are based on *F*, with *F* set to zero for negative *F*^2^. The threshold expression of *F*^2^ > σ(*F*^2^) is used only for calculating *R*-factors(gt) *etc*. and is not relevant to the choice of reflections for refinement. *R*-factors based on *F*^2^ are statistically about twice as large as those based on *F*, and *R*- factors based on ALL data will be even larger.

### Fractional atomic coordinates and isotropic or equivalent isotropic displacement parameters (Å^2^)

**Table:**

	*x*	*y*	*z*	*U*_iso_*/*U*_eq_	
O5	0.72771 (16)	0.24370 (18)	0.64625 (9)	0.0354 (3)	
C11	0.6615 (2)	0.3526 (2)	0.86815 (13)	0.0301 (4)	
O3	0.07767 (15)	0.25080 (16)	0.51676 (10)	0.0348 (3)	
O1	0.31588 (16)	0.45480 (17)	0.50248 (9)	0.0356 (3)	
N1	0.57765 (19)	0.33374 (19)	0.79210 (11)	0.0348 (4)	
N2	0.43215 (18)	0.42648 (18)	0.89530 (10)	0.0292 (3)	
O2	0.01088 (17)	0.3204 (2)	0.69454 (10)	0.0435 (4)	
C1	0.1920 (2)	0.2640 (2)	0.58171 (12)	0.0276 (4)	
C2	0.4434 (2)	0.3787 (2)	0.81175 (12)	0.0286 (4)	
C3	0.3703 (2)	0.4282 (2)	0.65804 (12)	0.0280 (4)	
C4	0.5722 (2)	0.41153 (19)	0.93338 (12)	0.0264 (4)	
C5	0.2540 (2)	0.4136 (2)	0.58515 (11)	0.0258 (4)	
C6	0.3142 (2)	0.3786 (2)	0.74770 (12)	0.0266 (4)	
C7	0.2462 (3)	0.2298 (2)	0.74082 (13)	0.0347 (4)	
C8	0.6284 (2)	0.4454 (2)	1.01561 (13)	0.0332 (4)	
C9	0.8684 (2)	0.3580 (2)	0.96474 (15)	0.0368 (5)	
C10	0.7779 (2)	0.4184 (2)	1.02923 (14)	0.0351 (4)	
C12	0.8121 (2)	0.3239 (2)	0.88366 (15)	0.0371 (5)	
C13	0.1264 (2)	0.2240 (2)	0.66954 (13)	0.0340 (4)	
O4	0.20679 (15)	0.47505 (16)	0.78330 (10)	0.0363 (3)	
H1	0.272 (2)	0.196 (2)	0.5665 (14)	0.029 (5)*	
H2	0.328 (3)	0.161 (3)	0.7259 (17)	0.045 (7)*	
H3	0.456 (3)	0.376 (2)	0.6402 (15)	0.033 (6)*	
H4	0.976 (3)	0.341 (2)	0.9775 (15)	0.038 (6)*	
H5	0.173 (3)	0.477 (2)	0.5973 (13)	0.026 (5)*	
H6	0.347 (3)	0.459 (3)	0.9186 (16)	0.042 (7)*	
H7	0.824 (3)	0.440 (3)	1.0846 (16)	0.041 (6)*	
H8	0.875 (3)	0.290 (3)	0.8364 (19)	0.061 (8)*	
H9	0.198 (3)	0.206 (3)	0.8007 (16)	0.042 (6)*	
H10	0.401 (3)	0.527 (3)	0.6663 (15)	0.043 (7)*	
H11	0.567 (3)	0.488 (3)	1.0588 (18)	0.045 (7)*	
H12	0.126 (3)	0.252 (3)	0.4704 (19)	0.058 (9)*	
H13	0.119 (3)	0.445 (3)	0.7643 (18)	0.054 (8)*	
H14	−0.069 (3)	0.299 (3)	0.6691 (18)	0.055 (8)*	
H15	0.393 (3)	0.403 (3)	0.4937 (18)	0.055 (8)*	
H16	0.088 (3)	0.123 (2)	0.6650 (14)	0.029 (6)*	
H17	0.745 (4)	0.156 (4)	0.661 (2)	0.074 (10)*	
H18	0.673 (4)	0.272 (4)	0.695 (2)	0.077 (10)*	

### Atomic displacement parameters (Å^2^)

**Table:**

	*U*^11^	*U*^22^	*U*^33^	*U*^12^	*U*^13^	*U*^23^
O5	0.0314 (7)	0.0459 (9)	0.0290 (7)	0.0014 (7)	0.0037 (6)	−0.0055 (6)
C11	0.0283 (9)	0.0381 (10)	0.0239 (9)	0.0032 (8)	−0.0024 (7)	−0.0007 (7)
O3	0.0279 (7)	0.0504 (8)	0.0260 (7)	−0.0044 (7)	−0.0035 (6)	−0.0059 (7)
O1	0.0302 (7)	0.0533 (9)	0.0235 (7)	−0.0023 (7)	−0.0003 (6)	0.0087 (6)
N1	0.0289 (8)	0.0517 (10)	0.0238 (8)	0.0084 (8)	−0.0033 (7)	−0.0078 (7)
N2	0.0233 (7)	0.0420 (9)	0.0224 (8)	0.0030 (7)	−0.0015 (6)	−0.0036 (7)
O2	0.0236 (7)	0.0740 (11)	0.0330 (8)	−0.0094 (7)	0.0042 (6)	−0.0140 (8)
C1	0.0251 (8)	0.0350 (9)	0.0226 (8)	0.0022 (8)	−0.0019 (7)	−0.0036 (7)
C2	0.0266 (9)	0.0364 (9)	0.0228 (9)	0.0013 (8)	0.0000 (7)	−0.0028 (7)
C3	0.0236 (8)	0.0362 (10)	0.0240 (9)	−0.0015 (8)	−0.0020 (7)	−0.0011 (7)
C4	0.0247 (8)	0.0320 (8)	0.0224 (9)	−0.0004 (7)	−0.0013 (7)	0.0006 (7)
C5	0.0223 (8)	0.0359 (9)	0.0192 (8)	0.0004 (8)	0.0002 (7)	0.0010 (7)
C6	0.0239 (8)	0.0362 (9)	0.0197 (8)	0.0016 (7)	−0.0004 (7)	−0.0047 (7)
C7	0.0413 (11)	0.0391 (10)	0.0239 (9)	−0.0045 (10)	−0.0038 (8)	0.0038 (8)
C8	0.0332 (9)	0.0429 (10)	0.0235 (9)	0.0001 (9)	−0.0019 (8)	−0.0028 (8)
C9	0.0264 (9)	0.0447 (11)	0.0394 (11)	0.0008 (9)	−0.0063 (8)	0.0066 (9)
C10	0.0341 (10)	0.0412 (10)	0.0300 (10)	−0.0045 (9)	−0.0109 (8)	0.0034 (8)
C12	0.0273 (9)	0.0486 (12)	0.0355 (11)	0.0068 (9)	0.0003 (8)	−0.0015 (9)
C13	0.0353 (10)	0.0387 (11)	0.0281 (10)	−0.0111 (9)	−0.0022 (8)	−0.0012 (8)
O4	0.0248 (7)	0.0507 (8)	0.0335 (8)	0.0058 (7)	−0.0027 (6)	−0.0141 (6)

### Geometric parameters (Å, °)

**Table:**

O5—H17	0.88 (3)	C3—C6	1.533 (3)
O5—H18	0.93 (4)	C3—H3	0.95 (2)
C11—C4	1.395 (3)	C3—H10	0.98 (3)
C11—N1	1.395 (2)	C4—C8	1.391 (3)
C11—C12	1.398 (3)	C5—H5	0.96 (2)
O3—C1	1.432 (2)	C6—O4	1.435 (2)
O3—H12	0.83 (3)	C6—C7	1.540 (3)
O1—C5	1.434 (2)	C7—C13	1.531 (3)
O1—H15	0.86 (3)	C7—H2	1.01 (3)
N1—C2	1.312 (2)	C7—H9	1.04 (2)
N2—C2	1.358 (2)	C8—C10	1.381 (3)
N2—C4	1.392 (2)	C8—H11	0.95 (3)
N2—H6	0.90 (3)	C9—C12	1.376 (3)
O2—C13	1.434 (3)	C9—C10	1.399 (3)
O2—H14	0.84 (3)	C9—H4	1.00 (2)
C1—C13	1.514 (3)	C10—H7	0.96 (2)
C1—C5	1.524 (3)	C12—H8	0.97 (3)
C1—H1	0.99 (2)	C13—H16	1.02 (2)
C2—C6	1.517 (3)	O4—H13	0.89 (3)
C3—C5	1.532 (2)		
			
H17—O5—H18	99 (3)	C1—C5—H5	108.0 (13)
C4—C11—N1	109.71 (16)	C3—C5—H5	108.7 (12)
C4—C11—C12	120.72 (19)	O4—C6—C2	105.52 (14)
N1—C11—C12	129.55 (19)	O4—C6—C3	111.29 (16)
C1—O3—H12	102.6 (19)	C2—C6—C3	109.00 (15)
C5—O1—H15	107.0 (19)	O4—C6—C7	110.09 (16)
C2—N1—C11	105.19 (16)	C2—C6—C7	110.33 (15)
C2—N2—C4	106.96 (16)	C3—C6—C7	110.49 (15)
C2—N2—H6	123.3 (16)	C13—C7—C6	111.08 (16)
C4—N2—H6	129.7 (16)	C13—C7—H2	109.1 (15)
C13—O2—H14	110 (2)	C6—C7—H2	108.6 (15)
O3—C1—C13	108.30 (15)	C13—C7—H9	109.1 (15)
O3—C1—C5	111.52 (15)	C6—C7—H9	107.7 (13)
C13—C1—C5	110.17 (15)	H2—C7—H9	111 (2)
O3—C1—H1	107.5 (12)	C10—C8—C4	116.39 (19)
C13—C1—H1	109.1 (12)	C10—C8—H11	122.5 (16)
C5—C1—H1	110.2 (12)	C4—C8—H11	121.1 (16)
N1—C2—N2	113.06 (17)	C12—C9—C10	121.11 (19)
N1—C2—C6	123.58 (17)	C12—C9—H4	119.5 (14)
N2—C2—C6	123.37 (16)	C10—C9—H4	119.4 (14)
C5—C3—C6	113.48 (15)	C8—C10—C9	122.24 (19)
C5—C3—H3	107.0 (14)	C8—C10—H7	120.5 (16)
C6—C3—H3	111.0 (14)	C9—C10—H7	117.3 (16)
C5—C3—H10	111.7 (14)	C9—C12—C11	117.5 (2)
C6—C3—H10	105.6 (14)	C9—C12—H8	122.3 (17)
H3—C3—H10	107.9 (19)	C11—C12—H8	120.0 (17)
C8—C4—N2	132.89 (18)	O2—C13—C1	110.91 (17)
C8—C4—C11	122.02 (17)	O2—C13—C7	107.13 (16)
N2—C4—C11	105.07 (16)	C1—C13—C7	110.40 (16)
O1—C5—C1	111.36 (15)	O2—C13—H16	111.8 (13)
O1—C5—C3	110.63 (14)	C1—C13—H16	107.9 (13)
C1—C5—C3	110.96 (15)	C7—C13—H16	108.7 (13)
O1—C5—H5	107.1 (12)	C6—O4—H13	105.7 (18)
			
C4—C11—N1—C2	−0.5 (2)	N1—C2—C6—C7	79.5 (2)
C12—C11—N1—C2	−179.1 (2)	N2—C2—C6—C7	−101.0 (2)
C11—N1—C2—N2	0.2 (2)	C5—C3—C6—O4	−71.7 (2)
C11—N1—C2—C6	179.75 (17)	C5—C3—C6—C2	172.34 (15)
C4—N2—C2—N1	0.2 (2)	C5—C3—C6—C7	50.9 (2)
C4—N2—C2—C6	−179.36 (17)	O4—C6—C7—C13	69.8 (2)
C2—N2—C4—C8	178.1 (2)	C2—C6—C7—C13	−174.09 (16)
C2—N2—C4—C11	−0.5 (2)	C3—C6—C7—C13	−53.5 (2)
N1—C11—C4—C8	−178.15 (18)	N2—C4—C8—C10	−178.0 (2)
C12—C11—C4—C8	0.6 (3)	C11—C4—C8—C10	0.4 (3)
N1—C11—C4—N2	0.6 (2)	C4—C8—C10—C9	−1.0 (3)
C12—C11—C4—N2	179.4 (2)	C12—C9—C10—C8	0.7 (3)
O3—C1—C5—O1	−59.24 (19)	C10—C9—C12—C11	0.3 (3)
C13—C1—C5—O1	−179.53 (15)	C4—C11—C12—C9	−0.9 (3)
O3—C1—C5—C3	177.05 (15)	N1—C11—C12—C9	177.6 (2)
C13—C1—C5—C3	56.76 (19)	O3—C1—C13—O2	−64.01 (19)
C6—C3—C5—O1	−177.00 (16)	C5—C1—C13—O2	58.20 (19)
C6—C3—C5—C1	−52.9 (2)	O3—C1—C13—C7	177.41 (16)
N1—C2—C6—O4	−161.58 (19)	C5—C1—C13—C7	−60.4 (2)
N2—C2—C6—O4	17.9 (2)	C6—C7—C13—O2	−61.7 (2)
N1—C2—C6—C3	−42.0 (3)	C6—C7—C13—C1	59.1 (2)
N2—C2—C6—C3	137.55 (19)		

### Hydrogen-bond geometry (Å, °)

**Table:**

*D*—H···*A*	*D*—H	H···*A*	*D*···*A*	*D*—H···*A*
N2—H6···O1^i^	0.90 (3)	2.11 (3)	2.983 (2)	164 (2)
O3—H12···O5^ii^	0.83 (3)	2.00 (3)	2.831 (2)	176 (3)
O4—H13···O2	0.89 (3)	1.87 (3)	2.660 (2)	148 (3)
O2—H14···O5^iii^	0.84 (3)	1.93 (3)	2.743 (2)	163 (3)
O1—H15···O3^iv^	0.86 (3)	2.21 (3)	3.066 (2)	172 (3)
O5—H17···O4^v^	0.88 (3)	1.96 (4)	2.827 (2)	169 (3)
O5—H18···N1	0.93 (4)	1.81 (4)	2.740 (2)	176 (3)

Symmetry codes: (i) −*x*+1/2, −*y*+1, *z*+1/2; (ii) *x*−1/2, −*y*+1/2, −*z*+1; (iii) *x*−1, *y*, *z*; (iv) *x*+1/2, −*y*+1/2, −*z*+1; (v) −*x*+1, *y*−1/2, −*z*+3/2.
